# Efficacy of Low-Pressure Inflation of Oversized Drug-Coated Balloon for Coronary Artery Disease

**DOI:** 10.1155/2020/6615988

**Published:** 2020-12-27

**Authors:** Katsumi Ueno, Norihiko Morita, Yoshinobu Kojima, Hiroki Kondo, Hiroshi Takahashi, Shingo Minatoguchi, Sho Higuchi, Yu Ando, Masayasu Esaki

**Affiliations:** ^1^Department of Cardiology, Matsunami General Hospital, Kasamatsu, Japan; ^2^Department of Medical Statistics, Fujita Health University School of Medicine, Toyoake, Japan; ^3^Department of Cardiology, Gifu University Graduate School of Medicine, Gifu, Japan; ^4^Department of Cardiology, Kizawa Memorial Hospital, Minokamo, Japan

## Abstract

**Objectives:**

This study sought to assess the efficacy of oversized drug-coated balloon (DCB) inflation at low pressure for the prevention of acute dissections and late restenosis.

**Background:**

The major limitation of DCB coronary angioplasty is the occurrence of severe dissection after inflation of DCB.

**Methods:**

Between 2014 and 2018, 273 consecutive patients were retrospectively studied. 191 lesions (154 patients) treated by oversized DCB inflation at low pressure (<4 atm, 2.4 ± 1.2 atm, DCB/artery ratio 1.14 ± 0.22; LP group) were compared with 135 lesions (119 patients) treated by the standard DCB technique (7.1 ± 2.2 atm, DCB/artery ratio 1.03 ± 0.16; SP group).

**Results:**

Although the lesions in the LP group were more complex than those in the SP group (smaller reference diameter (2.38 mm vs. 2.57 mm, *P*=0.011), longer lesions (11.7 mm vs. 10.5 mm, *P*=0.10), and more frequent use of rotational atherectomy (45.0% vs. 28.1%, *P*=0.003), there was no significant difference in the NHLBI type of dissections between the two groups (11.5%, 12.0%, 5.2% vs. 12.6%, 12.6%, 2.2% in type *A*, *B*, and *C*, *P*=0.61), and no bailout stenting was required. In 125 well-matched lesion pairs after propensity score analysis, the cumulative incidence of target lesion revascularization at 3 years was 4.5% vs. 7.0%, respectively (*P*=0.60). Late lumen loss (−0.00 mm vs. −0.01 mm, *P*=0.94) and restenosis rates (7.4% vs. 7.1%, *P*=1.0) were similar in both of the groups.

**Conclusion:**

The application of oversized DCB at low pressure is effective and feasible for preventing late restenosis comparative to the standard technique of DCB.

## 1. Introduction

Although coronary angioplasty with drug-coated balloons (DCB) has been found to have a low rate of target lesion revascularization comparative to those of drug-eluting stents (DES) [1, 2] and the complexity of the lesions treated with DCB has gradually increased, the major limitation of a DCB only strategy is the occurrence of severe dissections and subsequent stenting after inflation of DCB [3].

Diffuse, calcified, tortuous, and ostial lesions were reported as angiographical predictors of acute vessel closure after balloon coronary angioplasty [4–6]. Since the delivery of antiproliferative drug to the lesions with DCB is based on the mechanical inflation of a balloon, the application of DCB for such complex lesions should theoretically cause the same complications regardless of operator's experience [7]. In fact, some recent studies reported the occurrence of severe dissections and bailout stenting after inflation of DCB to be 10–15% [3, 8, 9].

On the other hand, inflation of oversized compliant balloons at low pressure prevents subsequent severe dissections after rotational atherectomy for complex lesions [10, 11]. Therefore, if the application of an oversized DCB at low pressure is effective for reducing dissections and late restenosis, DCB could safely be applied to complex lesions following successful lesion preparation.

Thus, in this study, a retrospective comparison of the acute and long-term results of low-pressure application of an oversized DCB against those of the standard technique of DCB after successful lesion preparation of ischemic de novo native coronary lesions was undertaken to assess the efficacy of a low-pressure inflation of an oversized DCB.

## 2. Methods

### 2.1. Study Population

Between April 2014 and December 2018, this retrospective study included all consecutive patients treated with DCB after successful lesion preparation at Matsunami General Hospital (Gifu, Japan).

Although selection of DCB strategy or stent strategy was left to the discretion of the operator, recommendation for DCB use at our institute follows the guidelines of the German Consensus Group on DCB use. In addition, complex anatomy such as ostial lesions, bifurcation lesions, and diffuse long lesions was also included.

Debulking with rotational atherectomy was considered for lesions which met one of the following criteria: (1) the lesion was not crossable by the smallest balloon or by intravascular ultrasound (IVUS) or optical frequency domain imaging (OFDI); (2) the lesion could not be dilated with a high-pressure balloon or a scoring balloon; or (3) the lesions had extensive intimal deposition of calcium assessed by an imaging catheter.

Patients with restenotic lesions, in-stent restenosis, bypass graft lesions, and ostial lesions of the side branch (if the main branch was stented) were excluded. All patients provided written informed consent. The study complied with the Declaration of Helsinki for investigation in human beings and was approved by the institutional ethics committee of our institution. The ethics committee of Matsunami General Hospital approved the research protocol (No. 472).

### 2.2. Interventional Procedure Details: Lesion Preparation

Patients were pretreated with daily doses of 100 mg of aspirin and 75 mg of clopidogrel or 3.75 mg of prasugrel. Heparin was administered to maintain an activated clotting time of >300 seconds during the procedure.

All cases were treated with IVUS guidance (View IT, 35 MHz, and AltaView, 40 MHz; Terumo Corp., Tokyo, Japan; or OptiCross: Boston Scientific, Natick, MA, USA) or OCT guidance (FastView^TM^ and LUNAWAVE^TM^, Terumo Corp.).

Lesion preparation was performed with a semi- or noncompliant balloon, scoring balloon (cutting balloon, NSE, Scoreflex), or debulking devices (rotational atherectomy or directional coronary atherectomy).

Even after successful lesion preparation, repeat angiography and intracoronary imaging were performed after a 15-minute wait in order to assess any acute recoil of the dilated lesions [12].

Successful lesion preparation was defined as a residual stenosis of <50%, and the thrombolysis in myocardial infarction (TIMI) flow was 3 without severe dissections (type *C*-*F* of the North American National Heart Lung and Blood Institute (NHLBI) classification [13]), although some type *C* dissections in our study were eligible for DCB therapy if it was considered to be therapeutically treatable based on imaging and safe after the 15-minute wait.

The way of applying DCB after successful lesion preparation, whether the standard technique or low-pressure inflation of oversized DCB, was left to the discretion of the operator.

### 2.3. Adjunctive Application of DCB: The Standard Technique

The only brand of DCB approved in Japan was the SeQuentPlease (B. Braun, Berlin, Germany). Although the standard pressure DCB technique (SP group) was performed according to the treatment recommendation of the German Consensus Group [14], the difference in the present study was that the sizing of DCB was determined with imaging devices from measured values of the proximal reference diameter (PRD) and the distal reference diameter (DRD) after lesion preparation. The DCB to artery ratio is close to 1 : 1, and DCB is inflated at nominal or slightly higher pressure.

If the difference between PRD and DRD was >0.5 mm, two DCBs, with sizing determined by the PRD and DRD, were inflated at nominal pressure. The balloon length exceeded the target lesion at both sides by at least 2 mm. Inflation time was at least >30 seconds.

### 2.4. Adjunctive Application of Oversized DCB at low pressure : The Low Pressure Technique (Offlabel Use)

**Figure 1:**
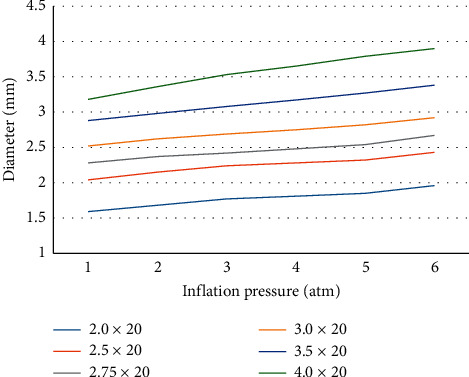
Measurements of balloon diameter (the SeQuentPlease) at each inflation pressure less than 6 atm (ex vivo). Three balloons of each size of the SeQuentPlease were measured with a digital caliper at each inflation pressure and mean values are shown. At 2-3 atm, the balloon diameter will be less by approximately 0.25–0.5 mm than the commercial size. At 1 atm, the diameter will be less than 0.5 mm of the commercial size.

**Figure 2:**
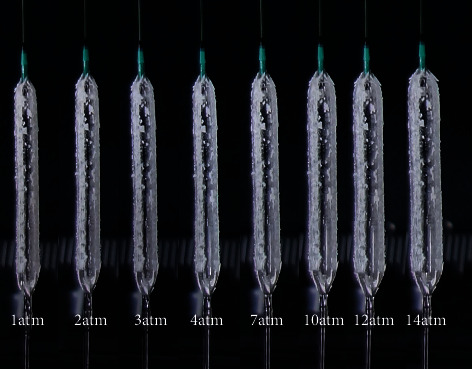
A 3.0 mm/20 mm-SeQuentPlease was inflated at each inflation pressure from 1 atm to 14 atm. The nominal pressure is 7 atm. Note that paclitaxel-iopromide complex is distributed evenly on the surface of the balloon at any inflating pressure.

Although various definitions of “low-pressure” (1 atmosphere (atm) [10] or less than 4 atm [11]) have been proposed, “low” pressure inflation (LP group) was defined as <4 atm due to DCB being considered to be less compliant than old compliant polyvinyl chloride balloons (Figures 1 and 2).

Oversized DCB (≥0.25 mm larger or 1.0-1.1 of the PRD) was used in order to ensure sufficient contact of the balloon surface with the vessel luminal wall during inflation at low pressure. Inflation time of at least >60 seconds was performed due to the time required to inflate the balloon to the full cylindrical form at low pressure (approximately 30 sec) than at nominal pressure (approximately 15 sec).

In applying a low-pressure inflation of DCB, several morphological factors such as lesion length and the difference between the PRD and DRD were taken into account. For example, in the case of tapered diffuse lesions with a larger difference between PRD and DRD (more than 0.5 mm), DCB by a 1 : 1 size or slightly larger size (ratio of DCB to artery: 1.1) of PRD was selected and inflated at low pressure.

In case of moderate or severely calcified plaque, tortuous lesions or ostial lesions, a low-pressure inflation of DCB was also considered.

When the reference diameter size was between commercially available sizes (i.e., such as 3.3 mm which is between 3.0 mm and 3.5 mm) (“between size”), a DCB of the larger diameter was selected and inflated at low pressure. The balloon length also exceeded the target lesion at both sides by at least 2 mm.

The operators confirmed the inflated shape of the balloon by angiography to ensure that the balloon was in full contact with the vessel luminal wall, as well as estimated by changes in the ST segment of ECG during inflation.

A residual stenosis <50% with TIMI 3 flow was defined to be successful.

### 2.5. Outcomes

The primary endpoint was acute outcomes (dissections and bailout stenting) after percutaneous coronary intervention (PCI) and clinically driven target lesion revascularization (TLR) at >12 months. The secondary endpoint was in segment late lumen loss (LLL) at > 6 months follow-up angiography. LLL was defined as post-PCI minimal lumen diameter (MLD) minus follow-up MLD. Acute gain (AG), which was defined as post-PCI-MLD minus pre-PCI-MLD, and angiographic restenosis (defined as percent diameter stenosis at follow-up >50%) were also investigated.

### 2.6. Quantitative Coronary Angiography

The angiograms before PCI, after PCI, and at follow-up were analyzed using the QAngio XA Version 7.3 (MEDIS Medical Imaging Systems BV, Leiden, the Netherlands).

### 2.7. Statistical Analysis

Continuous variables are presented as mean and standard deviation (SD) or median and interquartile range (IQR) and categorical variables as counts and percentages. Student's *t*-test and the chi-squared test were used for comparisons. A *P* value of <0.05 was considered statistically significant.

The cumulative incidence rates of TLR in the two groups were derived from Kaplan–Meier analyses, and the log-rank test was used to compare the differences between the groups. Cox proportional hazard models were used to compare the unadjusted outcomes between the groups, and the results are presented as hazard ratios (HR) with 95% confidence intervals (CI). To adjust for differences in baseline characteristics between the two procedures, propensity score matching was performed with a greedy matching algorithm. The matching algorithm used a multivariate logistic regression model that included baseline covariates with *P* < 0.05 in univariate analysis. All statistical analyses were performed by *R* software version 3.4.1(2017-06-30).

## 3. Results

### 3.1. Baseline Characteristics in the Crude Cohorts

**Figure 3:**
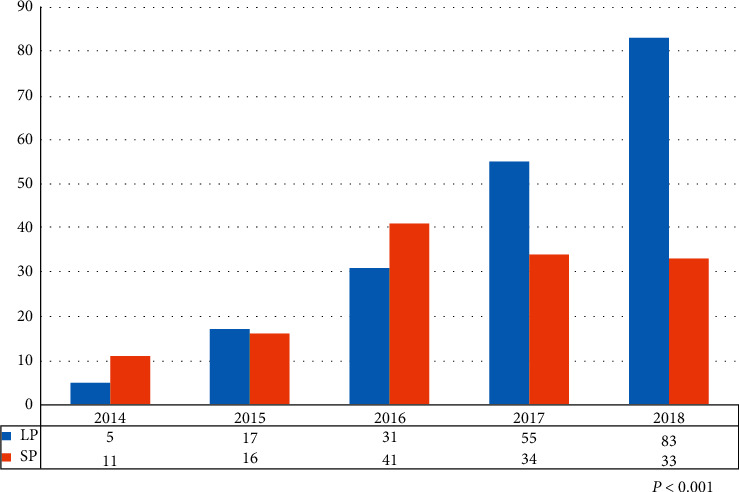
Relative distribution of low-pressure application of oversized DCB (LP group) and the standard pressure application of DCB (SP group).

The study population consisted of 191 lesions (154 patients) treated with oversized DCB inflated at low pressure (<4 atm) (LP group) and 135 lesions (119 patients) treated with the standard pressure technique of DCB (SP group). The median (IQR) follow-up was 787 (454, 1086) days. The relative distribution of the LP group and the SP group over the study period is shown in Figure 3. The ratio of the LP group to the SP group significantly increased over time (*P* < 0.001).

The baseline characteristics of the patients and the lesions are summarized in Table 1. There were no differences in baseline characteristics other than peripheral artery disease (PAD) (17.5% in the LP group vs. 6.8% in the SP group, *P*=0.007). In spite of no statistical difference, smoking (26% vs. 18.5%, *P*=0.15) and chronic kidney disease (26.6% vs. 18.5%, *P*=0.15) tended to be more frequent in the LP group than in the SP group.

Although there were no statistical differences in anatomical lesion morphology, type B2/C lesions in ACC/AHA lesion classification (81.6% vs. 74.0%, *P*=0.15) and visually assessed calcification (moderate/severe, 13.6%/35.1% vs. 14.8%/23.7%, *P*=0.16) tended to be more frequent in the LP group than in the SP group.

### 3.2. The Procedural and Follow-Up Angiographic Characteristics in the Crude Cohorts

The procedural and follow-up quantitative angiographic characteristics in the crude cohorts are summarized in Table 2. Use of the rotational atherectomy in the LP group was more frequent than in the SP group (45.0% vs. 28.1%, *P*=0.003), and angiographic burr-to-artery ratio tended to be higher in the LP group (0.76 ± 0.16 vs. 0.71 ± 0.17, *P*=0.11).

As a consequence of the method of this study, the inflation pressure in the LP group was significantly lower than that of the SP group (2.4 ± 1.2 atm vs. 7.1 ± 2.2 atm, *P* < 0.001), and the selected diameter of DCB to artery in the LP group was significantly larger than that of the SP group (1.14 ± 0.22 vs. 1.03 ± 0.16, *P* < 0.001). The inflation time in the LP group also tended to be longer (72.2 ± 39.8 sec vs. 64.8 ± 35.2 sec, *P* < 0.083).

QCA analysis before PCI showed that the reference diameter was significantly smaller in the LP group than in the SP group (2.38 ± 0.67 mm vs. 2.57 ± 0.66 mm, *P*=0.011), and the lesion length tended to be longer in the LP group than in the SP group (11.73 ± 6.87 mm vs. 10.54 ± 5.69 mm, *P*=0.10).

After PCI, MLD at post-PCI in the LP group was smaller than that of the SP group (1.74 ± 0.57 mm vs. 1.88 ± 0.49 mm, *P*=0.027), and acute gain (0.85 ± 0.47 mm vs. 0.94 ± 0.47 mm, *P*=0.091) in the LP group was smaller than that of the SP group. Moreover, percent diameter stenosis after PCI tended to be larger than that of the SP group (26.2 ± 11.9 mm vs. 24.1 ± 11.3 mm, *P*=0.11).

On the other hand, the occurrence of coronary dissection after inflation of DCB was similar in both groups (NHLBI A/B/C; 11.5%/12.0%/5.2% vs. 12.6%/12.6%/2.2%, *P*=0.61). There was no bailout stenting performed in either group after inflation of DCB.

Follow-up angiography was performed for 114 patients (74.0%) with 145 lesions (75.9%) in the LP group and 88 patients (73.9%) with 102 lesions (75.6%) in the SP group. MLD at follow-up (1.76 ± 0.62 mm vs. 1.89 ± 0.59 mm, *P*=0.094) and in percent diameter stenosis at follow-up (29.24 ± 17.55% vs. 25.77 ± 15.64%, *P*=0.11), LLL (-0.00 ± 0.42 mm vs. -0.01 ± 0.42 mm, *P*=0.86), and angiographic restenosis rate (13/145 (9.0%) vs. 10/102 (9.8%), *P*=0.83) were similar in both of the groups.

### 3.3. Follow-Up Angiographic Analysis of the Propensity Score Matching Groups

The propensity score was calculated from PAD, reference diameter, lesion length, and use of rotational atherectomy as covariates with *P* < 0.05 in univariate analysis as well as inflation time, since inflation time tended to be different (*P*=0.09) between the two groups and might have some influence on the long-term result.

In the propensity score-matched group (125 lesions in each group), there were no differences in all baseline characteristics of patients, lesions, and the procedure (Table 3).

As a result of the methodology, the inflation pressure in the LP group was lower than that of the SP group (2.5 ± 1.2 atm vs. 7.0 ± 2.1 atm, *P* < 0.001), and the selected diameter of DCB to artery was larger (1.11 ± 0.19 mm vs. 1.03 ± 0.17 mm, *P*=0.001). Inflation time in the LP group tended to be longer (72.3 ± 42.4sec vs. 64.4 ± 34.9 sec, *P*=0.11). However, the reference diameter (2.50 ± 0.69 mm vs. 2.55 ± 0.66 mm, *P*=0.58), lesion length (10.05 ± 5.18 mm vs. 10.63 ± 5.85 mm, *P*=0.40), the acute gain in luminal diameter (0.86 ± 0.52 mm vs. 0.91 ± 0.46 mm, *P*=0.41), and the percent diameter stenosis after PCI (26.5 ± 12.0% vs. 24.5 ± 11.1%, *P*=0.17) were similar between the two propensity-matched groups (Table 4).

At follow-up, MLD (1.85 ± 0.61 mm vs. 1.87 ± 0.57 mm, *P*=0.69), percent diameter stenosis (29.4 ± 15.5% vs. 26.0 ± 15.8%, *P*=0.13), LLL (−0.00 ± 0.40 mm vs. −0.01 ± 0.42 mm, *P*=0.94), and angiographic restenosis rate (7/95 (7.4%) vs. 7/99 (7.1%), *P*=1.0) were still similar in both propensity-matched groups (Table 4, at follow-up).

### 3.4. Clinical Follow-Up in the Crude Cohorts and in the Propensity-Matched Groups

**Figure 4:**
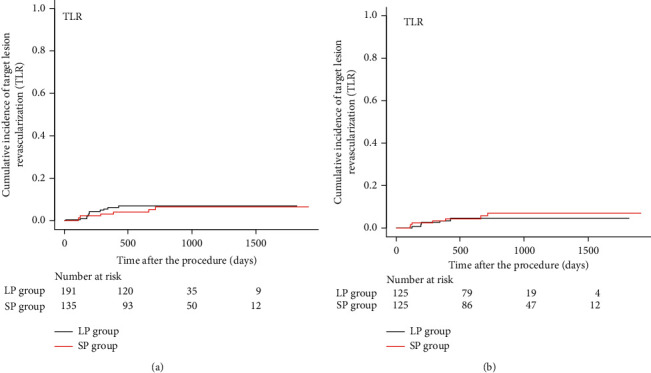
(a) Cumulative Kaplan–Meier estimates of the incidence of target lesion revascularization (TLR), using the crude cohorts of 191 lesions treated with oversized DCB at low pressure (LP group) and 135 lesions treated with DCB at standard pressure (SP group). (b) Cumulative Kaplan–Meier estimates of the incidence of target lesion revascularization (TLR), using the propensity-matched cohorts of 125 lesions treated with oversized DCB at low pressure (LP group) and 125 lesions treated with DCB at standard pressure (SP group).

The cumulative incidence rates of TLR at 1, 2, and 3 years were not significantly different between the LP and SP groups (6.2% vs. 3.1%, 6.9% vs. 6.5%, and 6.9% vs. 6.5%, respectively, *P*=0.65) (HR 0.81, 95% CI 0.32–2.00, *P*=0.65) (Figure 4(a)).

In the propensity score-matched groups, there were no significant differences in the cumulative incidence rates of TLR at 1, 2, and 3 years (3.5% vs. 3.4%, 4.5% vs. 7.0%, and 4.5% vs. 7.0%, respectively, *P*=0.60) (HR 1.36, 95% CI 0.43–4.30, *P*=0.60) (Figure 4(b)).

During the follow-up period, there were no definite cases of thrombosis in either group.

## 4. Discussion

The major limitation of the standard technique of a DCB only strategy is the occurrence of severe dissections and subsequent stenting immediately after inflation of DCB [3]. On the other hand, inflation of oversized compliant balloons at low pressure prevents severe dissections after rotational atherectomy for complex lesions [10, 11]. Therefore, if the application of oversized DCB at low pressure is effective for reducing severe dissections and long-term restenosis, DCB could safely be applied to complex lesions without the need for meticulous size selection once lesion preparation is successful.

To the best of our knowledge, this is the first report based on the efficacy of application of oversized DCB at low pressure (<4 atm). In order to assess efficacy of oversized DCB inflated at low pressure, a comparison was undertaken into the acute and long-term results of a low-pressure application of DCB (the LP group) with those of the standard pressure application of DCB (the SP group) for de novo native coronary lesions.

Our study had two major findings. First, although lesions treated with a low pressure DCB were more complex than those with the standard pressure DCB, there was no significant difference in the occurrence of dissections between the two groups and there was no bailout stenting. Several independent factors such as lesion length, tortuousity >45 degrees, thrombus, and the presence of calcification were reported as angiographical predictors of acute vessel closure after balloon angioplasty [4–6]. In the present study, the lesions in the LP group were obviously more complex (more diffuse, smaller, and more calcified requiring aggressive debulking) than those in the SP group. Although these results might suggest a safe application of DCB at low pressure for complex lesions which seem to be vulnerable to barotrauma after balloon inflation at nominal or higher pressure, the influence of “better” vessel preparation with rotational atherectomy on the acute results due to frequent use of rotational atherectomy in the crude cohorts as well as the propensity-matched groups is of important consideration. A prospective, randomized study would be needed to clarify the preventive effect of low-pressure inflation on the occurrence of coronary dissections.

Second, with regard to the long-term effect for preventing late restenosis, the application of DCB at low pressure was as effective as the standard application of DCB. After propensity matching, there were no significant differences in several parameters indicating the effect of antiproliferative drug such as TLR at 3 years, angiographic late lumen loss, percent diameter stenosis, and angiographic restenosis rate between the two groups. These results showed that the application of DCB at low pressure could also be as effective and feasible as the standard application of DCB for preventing late restenosis. It might be assumed that the diffusion of antiproliferative drugs into tissue might be caused due to contact of the balloon surface and luminal wall by its concentration gradient and not by pressure.

Of note, besides lesion morphology, the size of the balloon to artery should be of concern. The prospective, randomized trial instigated by Andreas R. Gruentzig, the greatest in PCI history, demonstrated oversized balloon dilation and multiple-lesion dilatation as procedural predictors of severe dissections and major complications after coronary balloon angioplasty [15]. The trial was halted as clinically important differences in acute complications emerged. They reported that the incidence of emergency surgery due to severe dissections was 1.7% when the balloon to artery ratio was <0.9, 3.1% when the ratio was 0.9–1.0, and 7.8% when the ratio was >1.1. Thus, the size of DCB plays as an important procedural factor.

Appropriate DCB size selection is problematic even with use of intracoronary imaging. One major factor hindering appropriate size selection is the existence of differences in diameter within the lesion itself (such as diffuse lesions or bifurcation lesions), having a significant difference in diameter between proximal and distal reference diameter (more than 0.25–0.5 mm). If a DCB sized by the proximal reference diameter is nominally inflated, overexpansion of the distal portion of the lesion will occur. Conversely, if a DCB sized in accordance with the distal reference diameter is used, underdilatation and incomplete apposition of the DCB to the proximal portion of the lesion would lead to incomplete delivery of antiproliferative drugs.

Furthermore, Gruentzig reported that multiple lesion dilatation is a risk factor for causing dissections. Multiple inflations are undertaken given that DCB inflation is performed as an additional inflation following lesion preparation. Dissections may therefore occur even when lesion preparation has been performed successfully for complex lesions, with greater potential given an oversized balloon for lesions with differences in diameter.

As explained by Lame's equation, a wall tension at a given pressure is increased as a multiple of the lumen size and is inversely proportional to the wall thickness. Therefore, the wall tension following successful lesion preparation, at any given pressure, is significantly increased due to a larger lumen and decreasing wall thickness after balloon dilatation or debulking [16]. Therefore, inflation at low pressure regardless of balloon sizing is safe, even following successful lesion preparation.

Finally, low-pressure inflation of oversized DCB was initially applied for severely calcified diffuse lesions in small vessels requiring rotational atherectomy [17]. Because of offlabel use, this approach was applied to a limited extent. As the long-term results of lesions initially treated with this technique were carefully observed, the indication of this technique has been extended not only to other forms of complex lesions but also to cases involving “between size” or “lesions with differing diameters” (Figure 3). This low-pressure technique could allow PCI operators to apply a DCB safely and easily without the need to precisely measure vessel size using intravascular imaging.

## 5. Study Limitations

There are several limitations in this study. First, only the SeQuentPlease (B. Braun, Berlin, Germany) was used. Second, the two procedures were assigned in a nonrandomized manner. Although we conducted propensity score matching to minimize the difference in patient characteristics, there may still be residual selection bias and confounding. Third, since this study was a retrospective, not double-blinded performed at a single center, a randomized study in a larger population is needed to define the acute and long-term effects of application of oversized DCB at low pressure. Fourth, the mechanism of delivery of the drug from the surface of the balloon to the lesion is still not clearly known. An in vitro study is needed to verify the results of our study.

## 6. Conclusion

The application of oversized DCB at low pressure is effective and feasible for preventing late restenosis comparative to the standard technique of DCB.

## Figures and Tables

**Table 1:** Baseline characteristics of patients and lesions in the crude cohorts.

Variables	LP group lesions (*n* = 191)	SP group lesions (*n* = 135)	*P* value
Number of patients	154	119	
Age (years)	69.0 ± 9.6	70.0 ± 10.0	0.44
Male (%)	103 (66.9)	93 (78.2)	0.89
Smoking (%)	40 (26.0)	22 (18.5)	0.15
Diabetes (%)	91 (59.1)	62 (52.1)	0.27
Hypertension (%)	112 (73.7)	88 (73.9)	1.00
Hyperlipidemia (%)	104 (67.5)	84 (70.6)	0.60
Chronic kidney disease (%)	41 (26.6)	22 (18.5)	0.15
Hemodialysis patients (%)	21 (13.6)	10 (8.4)	0.25
AMI (%)	6 (3.9)	9 (7.6)	0.29
Previous MI (%)	38 (24.7)	31 (26.1)	0.89
Previous CABG (%)	16 (10.4)	10 (8.4)	0.68
PAD (%)	27 (17.5)	8 (6.8)	0.007
Target vessel, *n* (%)			
LMT	5 (2.6)	3 (2.2)	0.92
LAD	82 (42.9)	57 (42.2)	
RCA	53 (27.7)	42 (31.1)	
LCX	51 (26.7)	33 (24.4)	
Lesion anatomy			
Type B2/C (%)	156 (81.6%)	100 (74.0%)	0.15
Ostial (%)	47 (24.6)	40 (29.6)	0.37
Bifurcation (%)	104 (54.5)	70 (51.9)	0.65
CTO (%)	17 (8.9)	11 (8.1)	0.84
Calcification (%) (visual assessment)			0.16
None	69 (36.1)	56 (41.5)	
Mild	29 (15.2)	27 (20.0)	
Moderate	26 (13.6)	20 (14.8)	
Severe	67 (35.1)	32 (23.7)	

AMI, acute myocardial infarction; B/A ratio, burr/artery ratio; CABG, coronary artery bypass graft surgery; CTO, chronic total occlusion; DCB, drug-coated balloon; DCB/A ratio, DCB/artery ratio; LAD, left anterior descending artery; LMT, left main trunk; LCX, left circumflex artery; MI, myocardial infarction; MLD, minimal lumen diameter; PAD, peripheral artery diseases; RCA, right coronary artery.

**Table 2:** Procedural and follow-up quantitative angiographic characteristics in the crude cohorts.

	LP group (*n* = 191)	SP group (*n* = 135)	*P* value
Number of patients	154	119	

Procedural devices			
DCA (%)	9 (4.7)	4 (3.0)	0.57
PTCRA (%)	86 (45.0)	38 (28.1)	0.003
B/A ratio	0.76 ± 0.16	0.71 ± 0.17	0.11

Drug-coated balloon			
DCB/A ratio	1.14 ± 0.22	1.03 ± 0.16	<0.001
Inflation pressure (atm)	2.4 ± 1.2	7.1 ± 2.2	<0.001
Inflation time (sec)	72.2 ± 39.8	64.8 ± 35.2	0.083

At PCI			
Reference diameter (mm)	2.38 ± 0.67	2.57 ± 0.66	0.011
Lesion length (mm)	11.73 ± 6.87	10.54 ± 5.69	0.10

MLD (mm)			
Preintervention (mm)	0.89 ± 0.45	0.94 ± 0.48	0.38
Postintervention (mm)	1.74 ± 0.57	1.88 ± 0.49	0.027

Percent diameter stenosis			
Preintervention (%)	62.9 ± 14.5	63.6 ± 15.1	0.69
Postintervention (%)	26.2 ± 11.9	24.1 ± 11.3	0.11

Acute gain (mm)	0.85 ± 0.47	0.94 ± 0.47	0.091

Type of dissection (NHLBI)			0.61
A	22 (11.5)	17 (12.6)	
B	23 (12.0)	17 (12.6)	
C	10 (5.2)	3 (2.2)	

Bailout stenting after DCB	0 (0)	0 (0)	
At follow-up			
Number of patients	114	88	
Angiography (%)	145 (75.9)	102 (75.6)	1.0
MLD (mm)	1.76 ± 0.62	1.89 ± 0.59	0.094
% diameter stenosis (%)	29.24 ± 17.55	25.77 ± 15.64	0.11
Late lumen loss (mm)	−0.00 ± 0.42	−0.01 ± 0.42	0.86
Restenosis (%)	13 (9.0)	10 (9.8)	0.83

MLD, minimal lumen diameter; SD, standard deviation; PCI, percutaneous coronary intervention; DCA, directional coronary atherectomy; PTCRA, percutaneous coronary rotational atherectomy.

**Table 3:** Baseline characteristics of patients, lesions, and procedure in the propensity-matched cohorts.

Variables	LP group lesions (*n* = 125)	SP group lesions (*n* = 125)	*P* value
Number of patients	108	113	
Age (years)	69.1 ± 9.5	70.1 ± 9.9	0.48
Male (%)	72 (66.7)	89 (78.8)	0.05
Smoking (%)	29 (26.9)	22 (19.5)	0.21
Diabetes (%)	60 (55.6)	60 (53.1)	0.79
Hypertension (%)	78 (72.2)	83 (73.5)	0.89
Hyperlipidemia (%)	72 (66.7)	80 (70.8)	0.56
Chronic kidney disease (%)	25 (23.1)	23 (20.4)	0.63
Hemodialysis patients (%)	12 (11.1)	10 (8.8)	0.66
AMI (%)	5 (4.6)	7 (6.2)	0.77
Previous MI (%)	25 (23.1)	31 (27.4)	0.54
Previous CABG (%)	8 (7.4)	10 (8.8)	0.81
PAD (%)	14 (13.0)	8 (7.1)	0.18
Target vessel, *n* (%)			0.92
LMT	3 (2.4)	3 (2.4)	
LAD	49 (39.2)	53 (42.4)	
RCA	37 (29.6)	38 (30.4)	
LCX	36 (28.8)	31 (24.8)	
Lesion anatomy			
Type B2/C (%)	96 (76.8)	92 (73.6)	0.90
Ostial (%)	36 (28.8)	39 (31.2)	0.78
Bifurcation (%)	68 (54.4)	68 (54.4)	1.00
CTO (%)	11 (8.8)	9 (7.2)	0.82
Calcification (%) (visual assessment)			0.96
None	53 (42.4)	50 (40.0)	
Mild	22 (17.6)	24 (19.2)	
Moderate	18 (14.4)	20 (16.0)	
Severe	32 (25.6)	31 (24.8)	
Procedural devices			
DCA (%)	8 (6.4)	4 (3.2)	0.38
PTCRA (%)	38 (30.4)	38 (30.4)	1.00
B/A ratio	0.75 ± 0.18	0.71 ± 0.17	0.31
Drug-coated balloon			
DCB/A ratio	1.11 (0.19)	1.03 (0.17)	0.001
Inflation pressure (atm)	2.5 (1.2)	7.0 (2.1)	<0.001
Inflation time (sec)	72.3 (42.4)	64.4 (34.9)	0.11

AMI, acute myocardial infarction; B/A ratio, burr/artery ratio; CABG, coronary artery bypass graft surgery; CTO, chronic total occlusion; DCB, drug-coated balloon; DCB/A ratio, DCB/artery ratio; LAD, left anterior descending artery; LMT, left main trunk; LCX, left circumflex artery; MI, myocardial infarction; MLD, minimal lumen diameter; PAD, peripheral artery diseases; RCA, right coronary artery.

**Table 4:** Procedural and follow-up quantitative angiographic characteristics in the matched cohorts.

	LP group (*n* = 125)	SP group (*n* = 125)	*P* value
Number of patients	108	113	

At PCI			
Reference diameter (mm)	2.50 ± 0.69	2.55 ± 0.66	0.58
Lesion length (mm)	10.05 ± 5.18	10.63 ± 5.85	0.40

MLD (mm)			
Preintervention	0.96 ± 0.45	0.94 ± 0.46	0.76
Postintervention	1.82 ± 0.60	1.85 ± 0.48	0.63

Percent diameter stenosis			
Preintervention (%)	61.6 ± 14.2	63.2 ± 14.5	0.39
Postintervention (%)	26.5 ± 12.0	24.5 ± 11.1	0.17

Acute gain (mm)	0.86 ± 0.52	0.91 ± 0.46	0.41

Type of dissection (NHLBI)			0.30
A	16 (12.8)	15 (12.0)	
B	11 (8.8)	16 (12.8)	
C	7 (5.6)	2 (1.6)	

Bailout stenting after DCB	0 (0)	0 (0)	

At follow-up			

Number of patients	81	87	

Angiography (%)	95 (76.0)	99 (79.2)	0.65
MLD (mm)	1.85 ± 0.61	1.87 ± 0.57	0.82
% diameter stenosis (%)	29.4 ± 15.5	26.0 ± 15.8	0.13
Late lumen loss (mm)	−0.00 ± 0.40	−0.01 ± 0.42	0.94
Restenosis (%)	7 (7.4)	7 (7.1)	1.00

DCB, drug-coated balloon; MLD, minimal lumen diameter; PCI, percutaneous coronary intervention.

## Data Availability

The data used to support the findings of this study are restricted by the Ethics Committee of Matsunami General Hospital in order to protect patient privacy and are available from the corresponding author for researchers who meet the criteria for access to confidential data.
